# A critical juncture for human rights in global health: Strengthening human rights through global health law reforms

**DOI:** 10.1371/journal.pgph.0002663

**Published:** 2023-12-08

**Authors:** Benjamin Mason Meier, Luciano Bottini Filho, Judith Bueno de Mesquita, Roojin Habibi, Sharifah Sekalala, Lawrence O. Gostin

**Affiliations:** 1 Department of Health Policy & Management, University of North Carolina at Chapel Hill, Chapel Hill, North Carolina, United States of America; 2 Helena Kennedy Centre for International Justice, Sheffield Hallam University, Sheffield, United Kingdom; 3 Essex Law School and Human Rights Centre, University of Essex, Colchester, United Kingdom; 4 Common Law Section, University of Ottawa, Ottawa, Ontario, Canada; 5 University of Warwick, Coventry, United Kingdom; 6 O’Neill Institute for National and Global Health Law, Georgetown University Law Center, Washington, DC, United States of America; PLOS: Public Library of Science, UNITED STATES; McGill University, CANADA

The 1948 Universal Declaration of Human Rights (UDHR) (Fig 1), establishing a human rights foundation under the United Nations (UN), has become a cornerstone of global health, central to public health policies throughout the world. As the world commemorates the 75th anniversary of the UDHR on 10 December, this “Human Rights Day” celebration arrives at a critical juncture for human rights in global health, raising an imperative for World Health Organization (WHO) reforms (Fig 2) to strengthen the right to health and health-related human rights.

**Fig 1:**
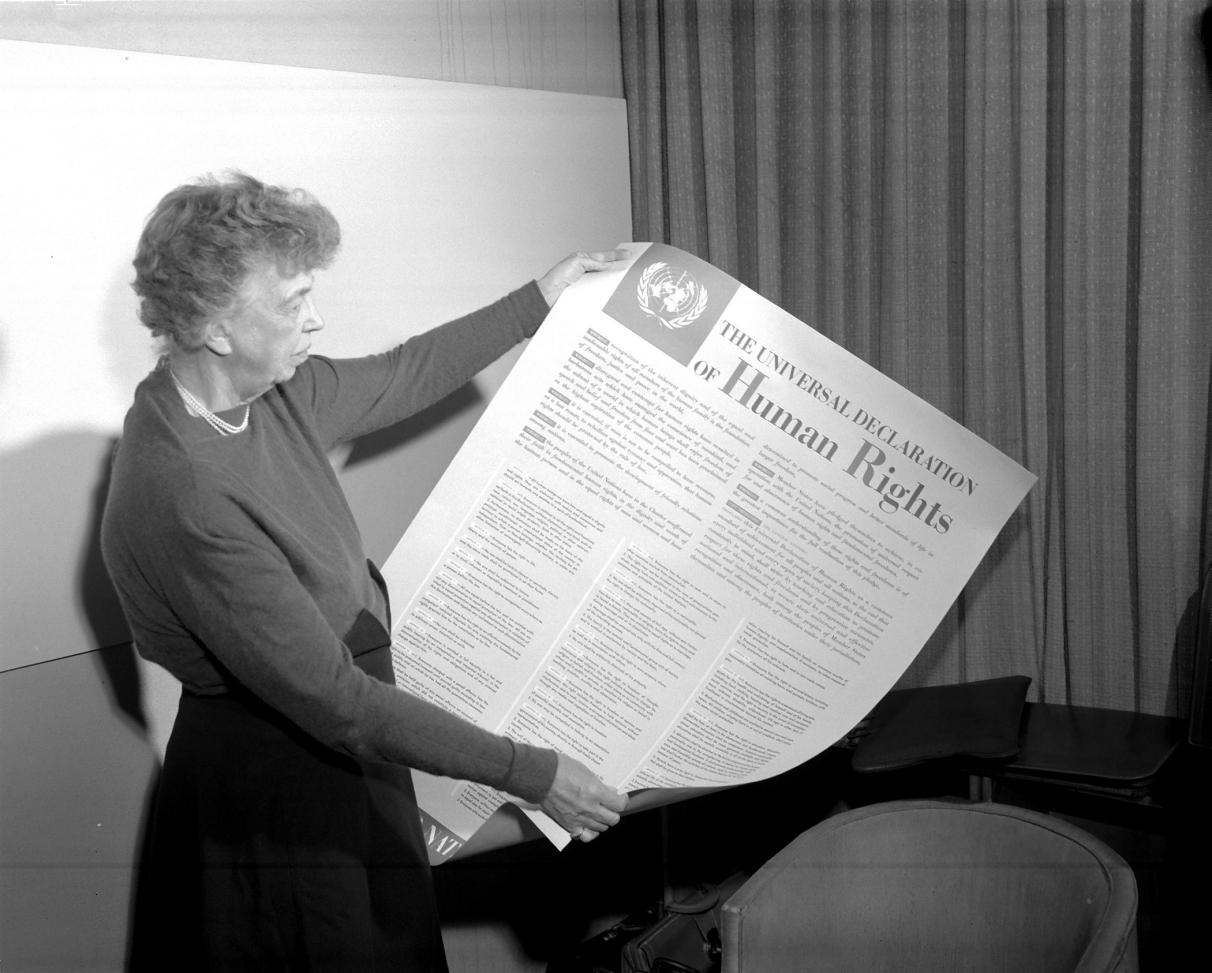
Commission on Human Rights Chair Eleanor Roosevelt exhibits the Universal Declaration of Human Rights (United Nations).

**Fig 2:**
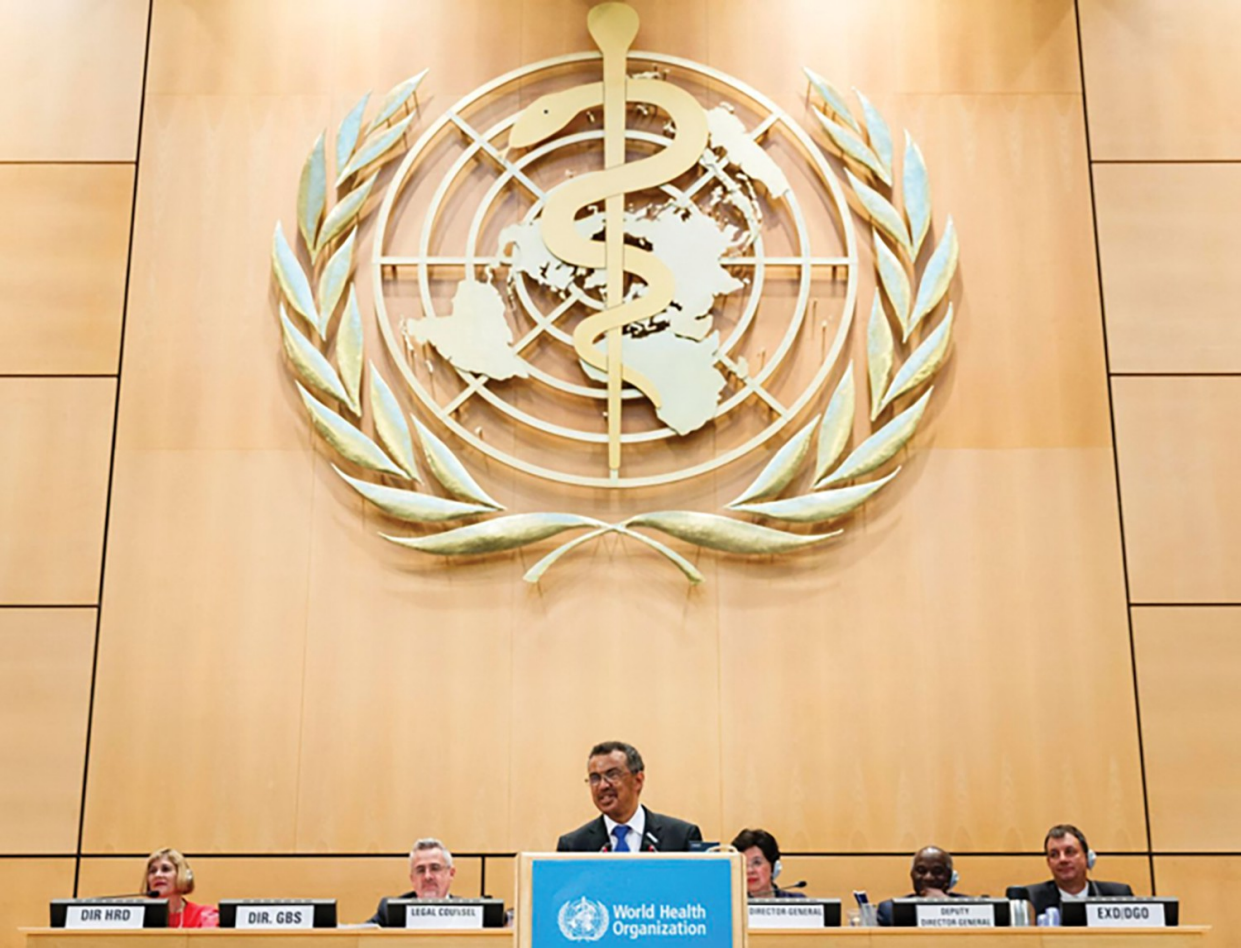
WHO Director-General Tedros Adhanom Ghebreyesus addresses the World Health Assembly (World Health Organization).

## Promising beginnings for global health and human rights

Out of the atrocities of World War II, states looked to advance human rights under international law, with the 1945 UN Charter elevating human rights as a principal basis of post-war governance. Establishing health governance under the UN, the 1946 WHO Constitution operationalized human rights for public health, proclaiming for the first time that “the enjoyment of the highest attainable standard of health is one of the fundamental rights of every human being” [1]. The UN General Assembly drew from these advancements in adopting the UDHR on 10 December 1948, enumerating a broad set of fundamental rights and declaring “a common standard of achievement for all peoples and all nations.” Public health was crucial to the UDHR, as states framed medical care and determinants of health as human rights:

Everyone has the right to a standard of living adequate for the health and well-being of himself and of his family, including food, clothing, housing and medical care and necessary social services… [2]

## Political challenges for human rights in global health

However, the advancement of human rights would face challenges in the years that followed. With the “Cold War” dividing the world, human rights became central to international divisions, with Western nations embracing civil and political rights and the Soviet Bloc favoring economic and social rights [3]. The “Universal Declaration” split along geopolitical lines, with the UN resorting to separate 1966 covenants: the International Covenant on Civil and Political Rights (ICCPR) and International Covenant on Economic, Social and Cultural Rights (ICESCR). Continuing Western opposition to socio-economic rights limited ICESCR codification of the “right of everyone to the enjoyment of the highest attainable standard of physical and mental health” [4].

Yet as colonized states gained independence and joined WHO governance, WHO embraced human rights anew as a basis to advance public health. WHO looked to the right to health in building political support for “primary health care” under the 1978 Declaration of Alma-Ata. Drawing from these efforts, the unfolding HIV/AIDS pandemic would give rise in the 1980s to a “health and human rights” movement. Civil society challenged government infringements on individual liberties in the early HIV/AIDS response, including discrimination against sexual minorities, as WHO came to recognize an “inextricable linkage” between public health and human rights–beginning in the equal rights of affected populations and extending to access to essential medicines [5].

At the turn of the millennium, the UN sought to interpret the right to health to reflect these modern public health principles, elaborating obligations to address “underlying determinants of health” [6]. WHO looked to establish these health-related rights under global health law [7]. As WHO Member States revised the International Health Regulations (IHR), amendments reflected human rights, with IHR (2005) requiring “full respect for the dignity, human rights and fundamental freedoms of persons” [8]. Human rights had become essential in responding to the health harms of a globalizing world; yet, an unprecedented pandemic would challenge human rights in global health.

## Rights violations in the pandemic response

As the COVID-19 pandemic unfolded, governments ignored scientific evidence, rejected global governance, and infringed human rights [9]. While the pandemic response initially held promise in bringing the world together to face a common challenge through universal rights, this hope rapidly gave way to ineffective and harmful measures. In violation of IHR obligations and fundamental rights, states introduced emergency measures that stoked discrimination, violated civil liberties, and restrained access to economic, health, and social needs [10].

Abandoning global solidarity in defiance of international obligations, wealthy nations instituted draconian border closures and discriminatory travel bans. These border closures came as governments in the Global North used their economic power to maintain control over essential medical countermeasures–ventilators, diagnostics, and personal protective equipment [11]. Undermining the right to health, these nationalist policies proved disastrous for poorer countries in the Global South, which faced underfunded health systems and weakened social protection programs.

These failures of global solidarity continued in global vaccination efforts. The international intellectual property regime proved incapable of ensuring vaccine equity to realize human rights. Even as the World Trade Organization provided concessions through limited patent waivers, legal obstacles endured in global efforts to ensure vaccine production and distribution [12]. With global governance dependent on the goodwill of donors and vaccine manufacturers, a two-tiered system emerged, as many in the Global South relied on inadequate donations while governments in the Global North hoarded vaccines, undermining justice in the pandemic response.

## Law reforms to revitalize rights in public health emergencies

These rights violations in a global health crisis have underscored the need to strengthen human rights obligations in future public health emergencies. Human rights can serve as a potent catalyst for government accountability and global solidarity. In many countries, advocates have realized significant health achievements through human rights, not only in preventing violations during the COVID-19 pandemic but also in advancing equity across health systems. These bottom-up movements have revitalized understanding and application of human rights under international law as a basis for public health.

Recognizing an imperative to align global health policy with human rights law, the Global Health Law Consortium and the International Commission of Jurists worked collaboratively through the COVID-19 pandemic to reach consensus on “Principles and Guidelines on Human Rights and Public Health Emergencies” [13]. These Principles clarify human rights standards applicable in the prevention of, preparedness for, response to, and recovery from such emergencies. In so doing, the Principles take an expansive view of “public health emergencies,” recognizing that safeguarding human rights remains essential to an effective and equitable public health emergency response.

Framing this emergency response in global governance, WHO Member States are undertaking negotiations to reform global health law, seeking amendments of the IHR and the development of a new Pandemic Accord; yet, diplomatic obstacles have raised uncertainty about whether the resulting legal instruments will prioritize human rights, equity, and accountability [14]. Looking beyond vague references to the “right to health,” advocates argue that transformative reforms must translate human rights principles into tangible obligations and mechanisms that facilitate accountability for human rights realization [15]. This human rights framework must be applied across global health policies, programmes, and practices, including critical areas such as access to medicines, intellectual property, health system financing, access and benefit-sharing, and One Health.

The coming negotiations, continuing amid rising international divisions, will determine how global governance meets future emergencies–and whether the world responds to global health threats without sacrificing our shared humanity. The future of human rights in global health will be established in these global health law reforms, but ultimately, the human rights movement must transcend this moment. For rights to be become a reality in health, realizing the promise of the UDHR, they must be mainstreamed across global health governance, ensuring that the rights of individuals are safeguarded in all global health efforts.
